# Simultaneous Determination of Aromatic Amines in Tattoo Ink by Gas Chromatography–Electron Ionization (GC-EI)–Mass Spectrometry (MS) and Tandem MS (MS/MS)

**DOI:** 10.3390/molecules31101623

**Published:** 2026-05-12

**Authors:** Eunyoung Shin, Hyebeen Kim, Jihye Choi, Minjae Kang, Juhui Shin, Sangwon Cha

**Affiliations:** Department of Chemistry, Dongguk University, Seoul 04620, Republic of Korea

**Keywords:** aromatic amines, tattoo inks, gas chromatography, mass spectrometry, tandem mass spectrometry

## Abstract

Aromatic amines (AAs) are potent carcinogens found in tattoo inks that pose significant health risks. Precise quantitative analysis is essential for safety. In this study, we developed and validated a robust method for the simultaneous quantification of 21 regulated AAs using both gas chromatography–electron ionization (GC-EI)–mass spectrometry (MS) and tandem MS (MS/MS). After optimizing separation conditions, MS-based selected ion monitoring (SIM) and MS/MS-based multiple reaction monitoring (MRM) were evaluated. While both methods were largely sufficient to ensure compliance with international safety thresholds, the MRM-based approach exhibited superior detection capability with comparable analytical accuracy and precision, providing a more effective tool for trace-level hazardous compound analysis. The developed MRM method was applied to eight commercial tattoo inks, identifying five AAs in five products. Notably, *o*-toluidine, *o*-anisidine, and 3,3′-dichlorobenzidine significantly exceeded the regulatory limit (5.0 mg/kg), particularly in green and yellow inks. The dual-capability GC-MS platform—combining high-performance MRM quantification with robust spectral confirmation—ensures the high throughput and analytical confidence necessary for regulatory compliance and public health protection.

## 1. Introduction

Tattooing has transitioned from a niche cultural practice to a widespread global phenomenon. This surge in popularity has heightened concerns regarding the chemical safety of tattoo inks, which are injected directly into the dermis and can persist in the body for a lifetime [1,2,3]. Recent epidemiological studies suggest that tattooed individuals may have a 21% higher risk of malignant lymphoma than non-tattooed individuals [2], with additional evidence indicating elevated risks of skin cancer and lymphoma, particularly for large tattoos [4]. These risks are thought to arise from the migration of ink particles to regional lymph nodes, where they accumulate and may induce chronic inflammation or carcinogenic processes [2,4,5]. Given these risks, the precise quantification of harmful substances in tattoo inks is critical to ensuring consumer safety [6,7].

Aromatic amines (AAs) are of particular toxicological concern as they are frequently used as intermediates in the production of synthetic azo dyes, which constitute the majority of pigments in yellow, orange, and red inks. The International Agency for Research on Cancer classifies several AAs, such as benzidine and *o*-toluidine, as Group 1 carcinogens, associated with increased risks of bladder and liver cancers [8]. In response to these health risks, global regulatory frameworks have become increasingly stringent. The European Chemicals Agency (ECHA) implemented REACH Regulation Annex XVII, Entry 75, restricting over 4000 hazardous substances and establishing a strict individual concentration limit of 5.0 mg/kg (0.0005% *w*/*w*) for regulated AAs [9]. Similarly, the Korean Ministry of Food and Drug Safety has recently reclassified tattoo inks under the sanitary products regulatory framework, introducing stricter safety requirements, including limits on AAs and other hazardous substances [10].

Previous studies established analytical foundations, but updated regulations require more refined methodologies. Early work by Hauri et al., employing liquid chromatography (LC)-mass spectrometry (MS) for the detection of carcinogenic AAs, primarily focused on ballpoint pen ink matrices [11]. Similarly, Lim and Shin developed a gas chromatography (GC)-MS approach to quantify AAs in tattoo dyes; however, the targeted species reflected the analytical priorities of that time and do not fully encompass the expanded list of primary AAs prioritized by current regulatory authorities [12]. Desmedt et al. later reported an LC-tandem MS (MS/MS) approach covering a broader range of regulated AAs; however, this method was largely designed for large-scale screening, with screening detection limits intended for identification rather than strict quantification [13]. More recently, Violi et al. conducted untargeted LC-MS/MS analysis of fifteen tattoo inks available in Australia, identifying two AAs listed in Regulation (EU) 2020/2081 [9]: toluidine in three samples and sulphanilic acid in nine samples. However, as this approach prioritized broad screening, it lacked the precise isomeric resolution and rigorous quantification necessary for full regulatory compliance [14]. As a result, additional sensitivity optimization may be required to meet the more stringent quantification criteria currently mandated by regulatory agencies.

We aimed to develop a GC–electron ionization (EI)–MS-based analytical method for the determination of 21 regulated AAs in tattoo inks. Our method focuses on free primary AAs specified in Appendix 13 of ECHA REACH Annex XVII, rather than those derived from the reductive cleavage of azo pigments. We compared the quantitative performance of selected ion monitoring (SIM) using GC–MS with multiple reaction monitoring (MRM) using GC–MS/MS, as previous studies have demonstrated that MRM can provide improved quantitation limits, accuracy, or precision compared to SIM, particularly in complex matrices such as biological fluids and food products (e.g., chocolate and margarine) [15,16]. EI-MS was chosen for its superior ability to provide structural confirmation in full-scan mode. Although quantitative analysis can be achieved using SIM or MRM, the ability to distinguish whether detected signals originate from target analytes or from matrix-derived interferences is critically important, especially in complex matrices such as tattoo inks. Accordingly, the optimized method was applied to the screening and quantification of 21 AAs in eight commercial tattoo ink products to evaluate its feasibility in routine compliance monitoring.

## 2. Results and Discussion

### 2.1. Optimization of Sample Preparation and GC Conditions

The target list of 21 AAs (Table 1) was established by selecting high-priority carcinogenic substances from the 31 AAs listed in the ECHA REACH Regulation Annex XVII, Entry 75, and its associated Appendix 13 (Table S1) [9]. These specific compounds were chosen to ensure the developed method meets the most stringent global safety requirements for substances of high concern in tattoo and permanent make-up inks.

Although the analytical framework was established with reference to ISO 14362-1:2017 [17,18], significant modifications were implemented to address the specific requirements of tattoo ink monitoring. Unlike the ISO standard, which identifies AAs released from azo colorants in textiles via reductive cleavage, our method focuses on the direct determination of free primary AAs within the ink matrix. This allowed for the omission of complex reductive cleavage and subsequent separation steps, enabling a more efficient liquid–liquid extraction (LLE)-based sample preparation. Furthermore, the target list was refined to include four additional compounds specified in Regulation (EU) 2020/2081—2,4-xylidine, 2,6-xylidine, 4-amino-3-fluorophenol, and 2-amino-6-ethoxynaphthalene—which are not covered by the ISO 14362-1:2017 target list.

The sample preparation procedure was optimized to reduce matrix effects while maintaining sufficient sensitivity for the determination of regulated AAs. A 50-fold dilution was selected because this dilution factor converts the regulatory level of 5.0 mg/kg to approximately 0.1 mg/L in the final extract, which remains above the instrumental LOQs obtained from standard solutions.

To effectively remove insoluble nano- to submicron-sized carbon black and pigment particles, hydrophilic and hydrophobic PTFE filters were tested, and a hydrophilic PTFE filter was selected over standard hydrophobic PTFE filters because the hydrophilic PTFE filter provided nearly transparent filtrates whereas the hydrophobic PTFE filter allowed a substantial amount of pigment particles to pass through (Figure S1). The enhanced filtration likely results from pigment adsorption onto the hydrophilic membrane network of the PTFE membrane, thereby reducing the effective pore size [19].

To assess whether the optimized filtration and extraction conditions caused significant analyte loss, recovery tests were conducted using different MeOH:DCM ratios (Table S2). Although previous studies have used LLE with MeOH or DCM as a single extraction solvent for AAs in ink products [11,12,13], neither single-solvent system provided satisfactory validation performance under our experimental conditions. Therefore, MeOH:DCM mixtures were evaluated to improve extraction efficiency and recovery. When a 1:9 (*v*/*v*) MeOH:DCM mixture was used, satisfactory recoveries ranging from 92% to 105% were achieved for the representative AAs. These results indicate that the target analytes were efficiently recovered without significant loss during the overall sample preparation procedure, including filtration.

**Figure 1:**
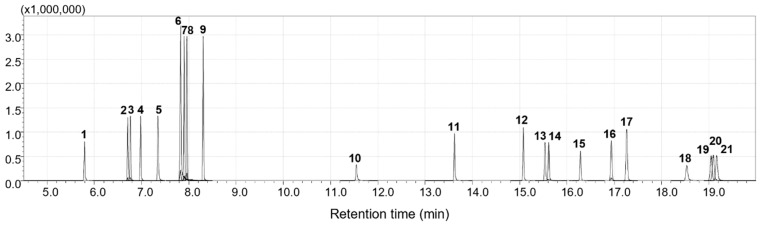
GC-MS chromatogram of target ions of 21 AAs with a DB-35 column acquired in SIM mode. Indicated numbers on peaks correspond to ID numbers of AAs listed in Table 1.

Following the sample preparation, GC separation conditions were optimized. The 35% diphenyl column (e.g., DB-35) recommended by ISO 14362-1:2017 was evaluated, along with the most widely used 5% diphenyl column (e.g., DB-5) and a 6% cyanopropylphenyl column (e.g., DB-624). As anticipated from the molecular characteristics of the analytes, the DB-35 column—where π-π interactions are predominant—exhibited superior resolution compared to the other columns (Figure 1 vs. Figure S2). The SIM conditions and retention time (RT) information for Figure 1 are summarized in Table 1, and other system suitability parameters such as theoretical plate count (*N*), capacity factor (*k*), and injection repeatability are presented in Table S3.

**Figure 2:**
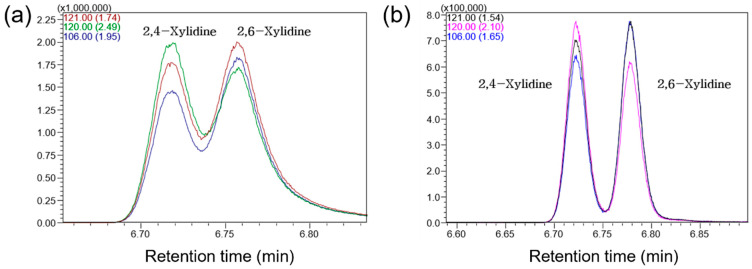
GC-MS SIM chromatograms for the target and reference ions of 2,4-xylidine and 2,6-xylidine using (**a**) splitless and (**b**) split (split ratio = 2:1) injection modes.

In addition, the sample injection mode was evaluated. During the initial phase of method development, splitless injection was employed to enhance sensitivity, rather than the split injection mode with a 15:1 split ratio suggested in ISO 14362-1:2017. When using the DB-35 column with splitless injection mode, most AAs were successfully separated. However, 4,4′-methylenebis(2-chloroaniline), 3,3′-dichlorobenzidine, and *o*-dianisidine, which elute around a RT of 19 min, were not fully resolved. Given that these three AAs possess distinct selected ions or MRM transitions, their partial co-elution was not expected to substantially affect quantitative accuracy. In contrast, the positional isomers 2,4-xylidine and 2,6-xylidine (RT ~6.7 min) required sufficient chromatographic resolution to enable reliable quantification. Accordingly, split injection mode was evaluated using various split ratios to improve peak separation while maintaining adequate sensitivity. As shown in Figure 2, a low split ratio of 2:1 significantly improved the resolution of these isomers. Due to the improved resolution, split mode injection exhibited superior linearity (*R*^2^) and precision (%RSD) in SIM mode quantification compared to splitless mode, while the limits of quantification (LOQs) remained comparable (Table 2). Therefore, a split ratio of 2:1 was selected as the optimal condition, providing the best compromise between sensitivity and chromatographic resolution while ensuring high reproducibility.

### 2.2. Optimization of MRM Conditions

**Figure 3:**
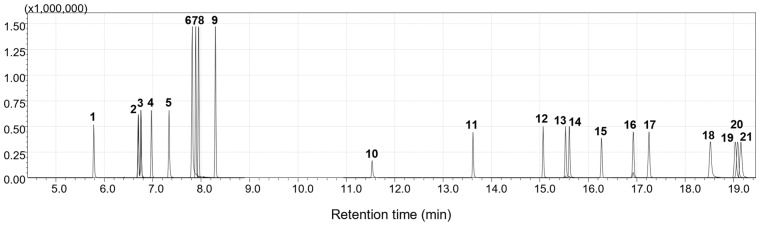
GC-MS/MS chromatogram of quantifier ions of 21 AAs acquired in MRM mode. Indicated numbers on peaks correspond to ID numbers of AAs listed in Table 3.

MRM parameters were optimized for 21 target compounds to maximize sensitivity and selectivity. For each analyte, the most intense transition was designated as the quantifier (target) ion, while two additional transitions were assigned as qualifiers to ensure reliable identification. Collision energies (CE) were systematically fine-tuned within a range of 3–45 V. GC-MRM chromatogram of 21 AAs is shown in Figure 3, and optimized MRM parameters are summarized in Table 3.

By selecting the most abundant transitions and validating the ion ratios (the intensity ratio between quantifier and qualifier ions), the optimized MRM conditions reliably facilitated both the quantification and identification of AAs. The final method was structured with 12 time segments over a total runtime of 18 min. This segmentation strategy ensured that transitions were monitored exclusively within their respective retention time windows, maintaining a minimum dwell time of 18.3 ms per transition. Consequently, background noise and matrix interferences were significantly reduced, leading to enhanced detection performance and analytical accuracy.

### 2.3. Comparison of Analytical Performance Between GC-MS and GC-MS/MS

The analytical performance of the developed SIM-based GC-MS and MRM-based GC-MS/MS methods was compared. For this evaluation, a black tattoo ink (designated ‘Black1’) verified to be free of AAs was used as a blank matrix to prepare matrix-matched calibration curves and AA-fortified samples. Matrix effects (ME) were evaluated by comparing the slopes of solvent-based calibration curves with those of matrix-matched curves. While most AAs showed acceptable ME within ±20% (Table S4), 4-amino-3-fluorophenol and a few others exhibited values approaching or exceeding this range, confirming the necessity of a matrix-matched calibration approach for accurate external quantification.

Table 4 summarizes the linear range, linearity (*R*^2^), and LOQ values for both acquisition modes. The MRM chromatogram of AAs at a concentration of 5 mg/kg in the ‘Black1’ matrix is shown in Figure S3. Calibration curves showed excellent linearity (*R*^2^ > 0.993) for all analytes. No significant mode-dependent degradation in linearity was observed. MRM mode significantly improved detection, yielding lower LOQs for 16 of the 21 analytes. A paired *t*-test confirmed this improvement to be statistically significant, with a mean LOQ reduction of 0.99 mg/kg (95% CI: 0.42–1.55 mg/kg; *p* < 0.002). Consequently, MRM allows for more reliable quantification at the trace levels required by stringent international regulations such as REACH.

The accuracy and precision of the methods were further evaluated through recovery experiments using the fortified ‘Black1’ matrix, with acceptance limits set at ±20% (Table 5). This criterion aligns with European residue-method validation guidelines (typical mean recovery of 70–120% and RSD ≤ 20%) [20]. While no statistically significant differences were observed in intra-day accuracy and precision, the MRM mode demonstrated superior inter-day robustness. Statistical analysis of the absolute deviations from the 100% recovery target revealed that the MRM mode (mean absolute error = 5.48%) was significantly closer to the theoretical target than the SIM mode (9.14%; *p* < 0.05).

A notable exception was observed for 4-amino-3-fluorophenol, which exhibited lower recoveries (62–84%) and significantly higher LOQs (4.9 mg/kg for MRM and 10.0 mg/kg for SIM) compared to other analytes. This behavior is likely associated with the intrinsic oxidative instability of its aminophenol moiety during sample preparation [21], combined with strong adsorption to the black tattoo ink matrix, particularly onto carbon black surfaces [22,23]. To improve the analytical performance for this specific compound without compromising the high-throughput efficiency of the established non-derivatized workflow, future optimization should prioritize targeted stabilization and compensation strategies. These include the addition of reducing agents (e.g., ascorbic acid or sodium sulfite) during extraction to minimize oxidative degradation, and the implementation of isotope dilution MS using structurally matched isotopically labeled internal standards to rigorously compensate for matrix effects and recovery variations near the 5.0 mg/kg regulatory threshold.

Based on these comprehensive evaluations, the MRM mode was selected as the optimal acquisition method due to its enhanced detection performance and was subsequently applied to the analysis of various other commercial tattoo ink samples.

The analytical significance of this study is further demonstrated through a comparative evaluation with previously reported methods. First, our target list is highly aligned with the 21 AAs prioritized by the REACH Regulation (EU) 2020/2081, whereas investigations conducted prior to this regulation [11,12] show a substantial mismatch with the current regulatory priority list. Second, the developed GC-EI-MS/MS method offers enhanced detection capability, with sample-based LOQs ranging from 0.4 to 4.9 mg/kg. These values represent a significant improvement over earlier LC-MS-based methods [11,13] and are comparable to or lower than the previous GC-MS report [12]. Most importantly, this study provides definitive chromatographic evidence for the resolution of critical positional isomers, such as 2,4- and 2,6-xylidine, which ensures high identification confidence.

### 2.4. GC-MS/MS Analysis of Tattoo Ink Samples

The developed MRM-based GC-MS/MS method was applied to the screening and quantification of 21 AAs in eight commercial tattoo ink products, including four black inks and one each of blue, green, red, and yellow inks. Quantitative analysis was performed using calibration curves established with the ‘Black1’ matrix. The applicability of this cross-matrix calibration was confirmed through recovery experiments across different ink colors, which produced values ranging from 80% to 120% for the majority of AAs (Table S5). Additionally, the slopes of calibration curves from various other matrix-matched standards exhibited deviations within an acceptable range of ±20% relative to the Black1-matrix-matched standards (Table S6 and Figure S4).

As summarized in Table 6, five different AAs were identified in five out of the eight products tested. Among these, three AAs—*o*-toluidine, *o*-anisidine, and 3,3′-dichlorobenzidine—were detected at concentrations exceeding the LOQ.

**Figure 4:**
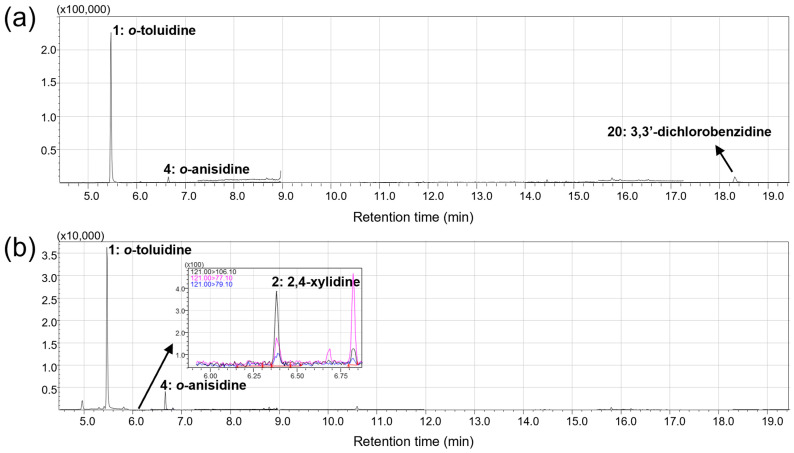
GC MRM chromatograms of (**a**) ‘Green’ and (**b**) ‘Yellow’ tattoo ink products acquired in MRM mode. The numbers indicated above the peaks correspond to the ID numbers of AAs listed in Table 6. Inset in panel (**b**) is magnified chromatogram of target and qualifier ions for 2,4-xylidine.

Notably, the concentrations of these analytes in certain samples significantly surpassed the individual concentration limit of 5.0 mg/kg mandated by the REACH Regulation Annex XVII, Entry 75. Specifically, *o*-toluidine and *o*-anisidine exceeded this regulatory threshold in both the ‘Green’ and ‘Yellow’ ink products, while 3,3′-dichlorobenzidine was found at non-compliant levels in the ‘Green’ ink. As observed in our study, several reports have identified *o*-toluidine and *o*-anisidine in green and yellow inks, often resulting from the cleavage of azo and amide bonds in organic pigments [5,7,11]. Figure 4 illustrates the GC MRM chromatograms for the green and yellow samples, showcasing the high selectivity of the method even in complex ink matrices. GC-MS chromatograms of these products in full scan mode are also provided in Figure S5.

A particularly high abundance of *o*-toluidine was observed in both ‘Green’ and ‘Yellow’ inks, with concentrations exceeding the initially established linear calibration range (>100 mg/kg). To rule out the possibility of co-eluting matrix interferences and to ensure unambiguous identification, a simultaneous full-scan MS acquisition was performed. As a representative case, the EI mass spectrum of *o*-toluidine obtained from the ‘Green’ sample at the specified RT showed an excellent correlation with that of the standard solution (Figure S6). A subsequent library search against the NIST 20 database yielded a similarity index exceeding 96%, providing unambiguous identification of the analyte. Potential carryover was also evaluated by injecting solvent blanks after these high-concentration samples. Although minor baseline elevations were observed, no identifiable peaks were detected at the target retention times, and the baseline fully stabilized after a single blank run.

## 3. Materials and Methods

### 3.1. Chemicals and Reagents

A total of 21 AA standards were purchased from various commercial suppliers. The following compounds were obtained from Sigma-Aldrich (St. Louis, MO, USA): 4-aminoazobenzene (CAS No. 60-09-3), *o*-aminoazotoluene (97-56-3), 2,4-xylidine (95-68-1), 2,6-xylidine (87-62-7), *o*-anisidine (90-04-0), 4-chloroaniline (106-47-8), 2-methoxy-5-methylaniline (120-71-8), 4-chloro-2-methylaniline (95-69-2), 2-methyl-5-nitroaniline (99-55-8), 4,4′-oxydianiline (101-80-4), 4,4′-diaminodiphenylmethane (101-77-9), 4,4′-diamino-3,3′-dimethyldiphenylmethane (838-88-0), *o*-tolidine (119-93-7), 4,4′-thiodianiline (139-65-1), 4,4′-methylenebis(2-chloroaniline) (101-14-4), 3,3′-dichlorobenzidine (91-94-1), *o*-dianisidine (119-90-4), and *o*-toluidine (95-53-4). Additionally, 2-amino-6-ethoxynaphthalene (293733-21-8) and 2,4,5-trimethylaniline (137-17-7) were sourced from LGC Standards (Teddington, UK), while 4-amino-3-fluorophenol (399-95-1) was obtained from Tokyo Chemical Industry (TCI, Tokyo, Japan). All reagents were of analytical grade or higher and used without further purification.

MeOH (99.9% purity for HPLC, GC) and DCM were purchased from Sigma-Aldrich. High-purity helium (He, 99.999%), high-purity argon (Ar, 99.999%) gases were purchased from Good Gas Co. (Pocheon, Republic of Korea). A hydrophilic PTFE membrane filter (Hyundai micro, Anseong, Republic of Korea) with a pore size of 0.45 μm was utilized. Eight tattoo ink products (four black, one red, one blue, one green, and one yellow inks) were obtained from online stores.

### 3.2. Preparation of Standards and Sample Preparation

Stock solution of 21 AA standards were prepared in MeOH at a concentration of 1000 mg/L. For the LLE of target compounds, 1 g of the tattoo ink sample was treated with 49 g of a MeOH/DCM mixture (1:9, *v*/*v*). The mixture was subjected to ultrasonic-assisted extraction at room temperature for 30 min. Following extraction, the resultant slurry was centrifuged at 10,000 rpm for 10 min. The supernatant was then filtered through a 0.45 μm hydrophilic PTFE filter prior to GC-MS analysis.

### 3.3. GC-MS and GC-MS/MS Analysis

GC analysis was performed using a Shimadzu GC-2030 gas chromatograph (Shimadzu, Kyoto, Japan) equipped with an AOC-20i Plus autosampler. Chromatographic separation was achieved on a DB-35MS UI capillary column (30 m × 0.25 mm i.d., 0.25 μm film thickness; Agilent J&W Scientific, Folsom, CA, USA). A 1 μL of the sample was injected in split mode (split ratio 2:1) with the injector temperature maintained at 250 °C. Helium (99.999%) served as the carrier gas at a constant flow rate of 1.0 mL/min. The oven temperature program was initiated at 65 °C (held for 0.5 min), increased to 220 °C at a rate of 25 °C/min (held for 0.5 min), then further raised to 240 °C at the same rate (25 °C/min, held for 0.5 min). Finally, the temperature increased to 280 °C at 25 °C/min and maintained for 8 min, resulting in a total run time of 22 min.

Mass spectrometric analysis was performed using a triple quadrupole mass spectrometer (GCMS-TQ8050 NX, Shimadzu, Kyoto, Japan) with the ion source and interface temperatures maintained at 200 °C and 250 °C, respectively. For the qualitative identification of the 21 AAs, the system was operated in Q3 scan mode and EI mass spectra were acquired in full scan mode across a range of *m*/*z* 50–500. Subsequent quantitative analysis was conducted using either selected ion monitoring (SIM) or multiple reaction monitoring (MRM) mode. For the MS/MS analysis in MRM mode, argon served as the collision gas for MS/MS fragmentation in MRM mode. To ensure unambiguous identification of the target analytes, the relative intensities of the quantifier and qualifier ions (ion ratios) were monitored. Following the Commission Decision 2002/657/EC and SANTE/11312/2021 guidelines, a maximum tolerance of ±20% for the ion ratios was applied for all identified compounds. The SIM and MRM conditions are summarized in Table 1 and Table 3, respectively.

### 3.4. Method Validation

Linearity of the method was evaluated by correlating peak areas with concentrations across at least five levels, ranging from 0.01 to 2 mg/L. The calibration curves were constructed using either solvent standard solutions or post-spiked matrix-matched standard solutions. Accounting for the 50-fold dilution during preparation, this range corresponds to 0.5–100 mg/kg in the original ink samples.

LOQs were determined using a fortified ‘Black1’ (a black tattoo ink confirmed to be AA-free) sample. The target concentration was 0.1 mg/L, except for 4-amino-3-fluorophenol, which was set at 0.4 mg/L (equivalent to 5 and 20 mg/kg in the sample, respectively). These values were calculated as 10σ/*m*, where σ represents the standard deviation of seven replicates and *m* is the calibration slope.

To evaluate the accuracy and precision of the analytical method, recovery experiments were performed using the ‘Black1’ tattoo ink matrix. The blank matrix was spiked with AA standards to achieve final concentrations of 10 mg/kg for most AAs, with the concentration for 4-amino-3-fluorophenol doubled to 40 mg/kg. Intra-day accuracy and precision were determined by analyzing three replicates within a single day. Inter-day accuracy and precision were assessed by repeating the same procedure over three consecutive days. Accuracy was expressed as the recovery percentage (%), while precision was calculated as % RSD.

Utilizing the obtained inter-day validation data, the expanded measurement uncertainty (*U*) was estimated following the top-down Nordtest approach [24] to ensure the method’s suitability for regulatory compliance near the 5.0 mg/kg REACH threshold. The calculation was performed using the following model:

$$U=k\times \sqrt{u_{rep}^{2}+u_{rec}^{2}+u_{sp}^{2}+u_{dil}^{2}}$$

where *k* is the coverage factor (*k* = 2), yielding a confidence level of approximately 95%; *u_rep_* is the uncertainty from reproducibility, directly derived from the inter-day precision (%RSD); *u_rec_* accounts for the method bias, calculated as |100 − recovery (%)| using the inter-day accuracy; and *u_sp_* represents the relative uncertainty of the spiking procedure, estimated at 0.1%. Furthermore, to adequately reflect the cumulative volumetric errors in samples requiring high-ratio dilutions (e.g., 200-fold and 1000-fold), an additional dilution uncertainty component (*u_dil_*) was introduced. While *u_dil_* was considered negligible for the standard 50-fold dilution, it was assigned a relative standard uncertainty of 1.5% for highly diluted samples, in accordance with the EURACHEM/CITAC guideline [25].

## 4. Conclusions

This study established a robust GC-EI-MS/MS method for the simultaneous quantification of 21 regulated AAs in tattoo inks. By optimizing the chromatographic conditions with a DB-35 column and a 2:1 split ratio, we achieved acceptable resolution for critical isomers. While both SIM and MRM modes met standard validation criteria, the MRM mode provided superior detection capability, lowering LOQs for 16 analytes and ensuring reliable detection below the 5.0 mg/kg REACH threshold.

The practical application of this method to commercial products revealed that five out of eight inks contained regulated AAs, with several samples significantly exceeding safety limits. While these results demonstrate the method’s utility, the limited sample size may affect the generalizability of the findings. Consequently, further studies with a broader range of formulations are warranted to confirm real-world applicability. Furthermore, a greenness assessment using the AGREE tool [26] yielded an overall score of 0.43 (Figure S7), reflecting a reasonable balance between high-performance analytical capability and environmental sustainability, despite the necessary use of organic solvents for optimal extraction efficiency.

Overall, this dual-capability platform—combining high-performance MRM quantification with robust spectral confirmation—offers a powerful and reliable tool for regulatory authorities to monitor hazardous substances in complex pigment matrices.

## Figures and Tables

**Table 1:** Target list of 21 aromatic amines (AAs), including their Chemical Abstracts Service (CAS) numbers, retention times (RTs), and selected ion monitoring (SIM) parameters.

ID No.	Analytes	CAS No.	RT (min)	Target Ion (*m*/*z*)	Ref. Ion 1 (*m*/*z*)	Ref. Ion 2 (*m*/*z*)
1	*o*-Toluidine	95-53-4	5.81	106	107	77
2	2,4-Xylidine	95-68-1	6.73	121	120	106
3	2,6-Xylidine	87-62-7	6.78	120	121	106
4	*o*-Anisidine	90-04-0	7.00	80	108	123
5	4-Chloroaniline	106-47-8	7.36	127	65	129
6	4-Amino-3-fluorophenol	399-95-1	7.83	127	98	52
7	2-Methoxy-5-methylaniline	120-71-8	7.91	122	137	94
8	2,4,5-Trimethylaniline	137-17-7	7.97	120	134	135
9	4-Chloro-2-methylaniline	95-69-2	8.32	106	141	77
10	2-Methyl-5-nitroaniline	99-55-8	11.56	152	77	79
11	2-Amino-6-ethoxynaphthalene	293733-21-8	13.64	158	187	130
12	4-Aminoazobenzene	60-09-3	15.10	92	197	120
13	4,4′-Oxydianiline	101-80-4	15.56	200	108	171
14	4,4′-Diaminodiphenylmethane	101-77-9	15.64	198	197	106
15	*o*-Aminoazotoluene	97-56-3	16.31	106	225	79
16	4,4′-Diamino-3,3′-dimethyldiphenylmethane	838-88-0	16.98	226	211	225
17	*o*-Tolidine	119-93-7	17.30	212	211	213
18	4,4′-Thiodianiline	139-65-1	18.58	216	184	215
19	4,4′-Methylenebis(2-chloroaniline)	101-14-4	19.11	231	266	195
20	3,3′-Dichlorobenzidine	91-94-1	19.15	252	254	253
21	*o*-Dianisidine	119-90-4	19.22	244	201	229

**Table 2:** Comparison of quantitative performance between splitless and split (2:1) injection modes for 2,4- and 2,6-xylidine.

ID No.	Analytes	Linearity (*R*^2^)		LOQ (mg/L) ^1^		Precision (%RSD) ^1^	
		Splitless	Split	Splitless	Split	Splitless	Split
2	2,4-Xylidine	0.990	0.997	0.02	0.02	3.4	1.5
3	2,6-Xylidine	0.990	0.998	0.03	0.03	3.8	1.9

^1^ LOQ (mg/L in solution) and precision values listed in this table were estimated against the standard solution. LOQ values were calculated as 10σ/*m*, where σ is the standard deviation of replicate measurements (*n* = 7) at 50 ppb and *m* is the slope of the calibration curve.

**Table 3:** Optimized MRM transitions and collision energies (CEs) for aromatic amines in elution order.

ID No.	Analytes	Quantifier Ion		Qualifier Ion 1		Qualifier Ion 2	
		Transition (*m*/*z*)	CE (V)	Transition (*m*/*z*)	CE (V)	Transition (*m*/*z*)	CE (V)
1	*o*-Toluidine	107 > 77	27	107 > 79	21	107 > 89	27
2	2,4-Xylidine	121 > 106	15	121 > 79	27	121 > 77	27
3	2,6-Xylidine	121 > 77	27	121 > 106	21	121 > 79	21
4	*o*-Anisidine	123 > 108	12	123 > 80	21	123 > 53	30
5	4-Chloroaniline	127 > 65	27	127 > 92	15	127 > 100	9
6	4-Amino-3-fluorophenol	127 > 98	21	127 > 52	24	127 > 79	15
7	2-Methoxy-5-methylaniline	137 > 122	12	137 > 94	21	137 > 77	27
8	2,4,5-Trimethylaniline	135 > 120	15	135 > 77	30	135 > 91	27
9	4-Chloro-2-methylaniline	141 > 106	12	141 > 77	30	141 > 79	21
10	2-Methyl-5-nitroaniline	152 > 106	12	152 > 77	27	152 > 79	21
11	2-Amino-6-ethoxynaphthalene	187 > 158	12	187 > 130	24	187 > 103	33
12	4-Aminoazobenzene	197 > 92	15	197 > 120	9	197 > 65	30
13	4,4′-Oxydianiline	200 > 108	21	200 > 80	30	200 > 171	18
14	4,4′-Diaminodiphenylmethane	198 > 182	12	198 > 106	12	198 > 180	30
15	*o*-Aminoazotoluene	225 > 160	15	225 > 134	9	225 > 79	27
16	4,4′-Diamino-3,3′-dimethyldiphenylmethane	226 > 211	15	226 > 195	27	226 > 196	24
17	*o*-Tolidine	212 > 196	24	212 > 180	30	212 > 169	30
18	4,4′-Thiodianiline	216 > 184	18	216 > 156	21	216 > 124	27
19	4,4′-Methylenebis(2-chloroaniline)	266 > 231	15	266 > 195	21	266 > 229	12
20	3,3′-Dichlorobenzidine	253 > 226	15	253 > 155	30	253 > 216	18
21	*o*-Dianisidine	244 > 201	18	244 > 158	33	244 > 186	27

**Table 4:** Comparison of method validation parameters (linear range, *R*^2^, and LOQs) for aromatic amines between SIM-based GC-MS and MRM-based GC-MS/MS methods.

ID No.	Analytes	Linear Range (mg/kg)		Linearity (*R*^2^)		LOQ in Sample (mg/kg)	
		SIM	MRM	SIM	MRM	SIM	MRM
1	*o*-Toluidine	2.0–100	1.0–100	0.999	0.999	1.6	0.8
2	2,4-Xylidine	2.0–100	2.0–100	0.996	0.999	1.1	1.1
3	2,6-Xylidine	5.0–100	5.0–100	0.998	0.997	2.8	1.7
4	*o*-Anisidine	1.0–100	0.5–100	0.999	0.999	0.6	0.5
5	4-Chloroaniline	1.0–100	1.0–100	0.998	0.997	0.8	0.8
6	4-Amino-3-fluorophenol	10–100	5.0–100	0.996	0.999	10.0	4.9
7	2-Methoxy-5-methylaniline	5.0–100	2.0–100	0.998	0.995	2.5	1.4
8	2,4,5-Trimethylaniline	5.0–100	2.0–100	0.998	0.997	2.5	1.8
9	4-Chloro-2-methylaniline	2.0–100	2.0–100	0.997	0.996	1.6	1.0
10	2-Methyl-5-nitroaniline	5.0–100	5.0–100	0.995	0.997	4.2	2.5
11	2-Amino-6-ethoxynaphthalene	5.0–100	5.0–100	0.996	0.995	1.7	1.8
12	4-Aminoazobenzene	1.0–100	1.0–100	0.997	0.998	0.5	0.6
13	4,4′-Oxydianiline	5.0–100	5.0–100	0.995	0.993	2.8	2.9
14	4,4′-Diaminodiphenylmethane	5.0–100	5.0–100	0.996	0.997	3.0	2.3
15	*o*-Aminoazotoluene	5.0–100	2.0–100	0.995	0.993	2.0	0.7
16	4,4′-Diamino-3,3′-dimethyldiphenylmethane	5.0–100	2.0–100	0.996	0.997	2.7	1.1
17	*o*-Tolidine	1.0–100	0.5–100	0.996	0.995	0.6	0.4
18	4,4′-Thiodianiline	5.0–100	2.0–100	0.993	0.994	3.8	2.0
19	4,4′-Methylenebis(2-chloroaniline)	5.0–100	2.0–100	0.995	0.996	2.4	1.9
20	3,3′-Dichlorobenzidine	2.0–100	1.0–100	0.998	0.996	1.2	0.7
21	*o*-Dianisidine	5.0–100	2.0–100	0.996	0.996	4.8	1.7

**Table 5:** Intra-day and inter-day accuracy and precision for 21 AAs in black tattoo ink using SIM and MRM modes.

ID No.	Analytes	Intra-Day (*n* = 3)				Inter-Day (*n* = 3)			
		Accuracy (%)		Precision (%)		Accuracy (%)		Precision (%)	
		SIM	MRM	SIM	MRM	SIM	MRM	SIM	MRM
1	*o*-Toluidine	106	101	3.4	1.8	91	101	14.4	11.9
2	2,4-Xylidine	103	110	3.7	3.4	98	110	9.0	9.7
3	2,6-Xylidine	97	102	7.8	5.0	87	105	10.0	7.4
4	*o*-Anisidine	95	99	3.7	1.9	88	99	10.9	11.7
5	4-Chloroaniline	97	87	3.3	2.4	100	105	8.5	8.4
6	4-Amino-3-fluorophenol	68	77	3.1	3.7	62	84	8.1	10.4
7	2-Methoxy-5-methylaniline	96	100	4.7	6.5	93	95	10.8	12.6
8	2,4,5-Trimethylaniline	93	111	3.0	4.5	87	96	4.0	8.9
9	4-Chloro-2-methylaniline	95	94	3.6	5.5	92	98	6.4	8.9
10	2-Methyl-5-nitroaniline	89	99	11.8	7.2	94	106	3.2	5.5
11	2-Amino-6-ethoxynaphthalene	101	109	5.4	1.8	97	93	18.4	14.3
12	4-Aminoazobenzene	107	105	3.9	3.4	101	109	13.4	9.2
13	4,4′-Oxydianiline	98	97	6.0	9.1	98	96	14.7	9.9
14	4,4′-Diaminodiphenylmethane	82	87	1.2	5.6	84	87	4.2	8.3
15	*o*-Aminoazotoluene	106	100	7.7	8.4	93	105	9.3	17.7
16	4,4′-Diamino-3,3′-dimethyldiphenylmethane	92	83	9.1	6.3	87	96	12.1	14.6
17	*o*-Tolidine	84	93	2.3	5.6	86	103	8.3	4.6
18	4,4′-Thiodianiline	101	108	8.1	5.6	92	98	9.3	5.5
19	4,4′-Methylenebis(2-chloroaniline)	102	108	2.0	2.6	94	106	12.3	9.8
20	3,3′-Dichlorobenzidine	102	98	2.7	4.6	100	104	10.3	4.4
21	*o*-Dianisidine	105	108	5.2	5.1	86	97	8.4	6.6

**Table 6:** Concentrations of AAs found in various tattoo ink products.

ID No. ^2^	Analytes	Concentration ± *U* ^1^ (mg/kg, *n* = 5)							
		Black1 ^2^	Black2	Black3	Black4	Blue	Green	Red	Yellow
1	*o*-Toluidine	- ^3^	-	-	-	1.61 ± 0.38	494 ± 119 ^5^	<LOQ	138 ± 33 ^5^
2	2,4-Xylidine	-	-	-	-				<LOQ
4	*o*-Anisidine	-	-	-	-		3.0 ± 0.7		13 ± 3
10	2-Methyl-5-nitroaniline	-	-	<LOQ ^4^	-				
20	3,3′-Dichlorobenzidine	-	-	4.0 ± 0.5	-	2.0 ± 0.2	7 ± 0.8		

^1^ *U*: expanded measurement uncertainty calculated using Nordtest method. ^2^ AAs not listed in this table were not detected in any of the analyzed tattoo ink products. ^3^ ‘-’: Not detected. ^4^ <LOQ: Analyte detected, but the concentration was below the LOQ. ^5^ To determine *o*-toluidine, Green and Yellow tattoo inks were diluted 1000-fold and 200-fold, respectively.

## Data Availability

The original contributions presented in this study are included in the article/Supplementary Materials. Further inquiries can be directed to the corresponding author.

## Supplementary Materials

The following supporting information can be downloaded at: https://www.mdpi.com/article/10.3390/molecules31101623/s1, Table S1: List of 31 primary AAs listed in ECHA REACH Regulation Annex XVII, Entry 75, Appendix 13 and their inclusion in the current study; Table S2: Recovery rates of three target AAs across different extraction solvent compositions, determined by GC–MS/SIM analysis of matrix-spiked ‘Black1’ samples; Table S3: System suitability parameters for the 21 target AAs, including theoretical plate count (*N*), capacity factor (*k*), and injection repeatability (%RSD of peak area, *n* = 3); Table S4: Evaluation of ME for AAs in the ‘Black1’ tattoo ink sample; Table S5: Evaluation of cross-matrix applicability: Recoveries (%) of AAs in various colored tattoo inks; Table S6: Comparative evaluation of relative ME for AAs in various colored tattoo inks relative to the ‘Black1’ matrix; Figure S1: Visual comparison of filtrates from ‘Black1’ tattoo ink (50-fold diluted with 1:9 MeOH:DCM) after filtration through a hydrophilic PTFE filter (left) and a hydrophobic PTFE filter (right); Figure S2: GC-MS chromatogram of target ions of 21 AAs with a DB-624 column acquired in SIM mode; Figure S3: MRM chromatograms for AAs at a concentration of 5 mg/kg in the ‘Black1’ matrix; Figure S4: Standard and matrix-matched calibration curves of ten representative AAs across different colored tattoo ink matrices (Black1, Red, Blue, Yellow, and Green) and solvent standard; Figure S5: GC-MS chromatograms of (a) ‘Green’ and (b) Yellow’ tattoo inks acquired in full scan mode; Figure S6: (a) EI mass spectrum of the *o*-toluidine standard solution and (b) EI mass spectrum extracted from the GC-MS analysis of the ‘Green’ tattoo ink sample; Figure S7: AGREE-based greenness assessment of the proposed method.

## Abbreviations

The following abbreviations are used in this manuscript:

AA	Aromatic amine
CAS	Chemical Abstracts Service
CE	Collision energy
DCM	Dichloromethane
ECHA	European Chemicals Agency
GC	Gas chromatography
LC	Liquid chromatography
LLE	Liquid–liquid extraction
LOQ	Limit of quantification
ME	Matrix effect
MeOH	Methanol
MS	Mass spectrometry
MS/MS	Tandem mass spectrometry
MRM	Multiple reaction monitoring
RSD	Relative standard deviation
SIM	Selected ion monitoring
