# Supplementary material for: Simultaneous Determination of Aromatic Amines in Tattoo Ink by Gas Chromatography–Electron Ionization (GC-EI)–Mass Spectrometry (MS) and Tandem MS (MS/MS)
Source: Molecules. 2026 May 12;31(10):1623. doi: 10.3390/molecules31101623 (PMC13209397; doi:10.3390/molecules31101623)
Supplement: Supplementary file 1 [file molecules-31-01623-s001.zip › molecules-4276390-supplementary.pdf]

Supporting Information

Simultaneous Determination of Aromatic Amines in Tattoo Ink by Gas Chromatography-Electron Ionization (GC-EI)-Mass Spectrometry (MS) and -Tandem MS (MS/MS)

Eunyoung Shin[1,†], Hyebeen Kim[1,†], Jihye Choi[1], Minjae Kang[1], Juhui Shin[1], and Sangwon Cha[1,*]

[1]Department of Chemistry, Dongguk University, Seoul 04620, Republic of Korea

† These authors contributed equally to this work.

*Correspondence: chasw@dongguk.edu; Tel.: +82-2-2260-8907 (S.C.)

Table S1. List of 31 primary aromatic amines listed in ECHA REACH Regulation Annex XVII, Entry 75, Appendix 13 and their inclusion in the current study

No.	Substance Name	EC No.	CAS No.	Analyte in this study (Y/N)
1	*o*-anisidine	201-963-1	90-04-0	Y
2	*o*-toluidine	202-429-0	95-53-4	Y
3	3,3'-dichlorobenzidine	202-109-0	91-94-1	Y
4	4-methyl-*m*-phenylenediamine	202-453-1	95-80-7	N
5	4-chloroaniline	203-401-0	106-47-8	Y
6	5-nitro-*o*-toluidine (2-methyl-5-nitroaniline)	202-765-8	99-55-8	Y
7	3,3'-dimethoxybenzidine (*o*-dianisidine)	204-355-4	119-90-4	Y
8	4,4'-bi-*o*-toluidine (*o*-tolidine)	204-358-0	119-93-7	Y
9	4,4'-thiodianiline	205-370-9	139-65-1	Y
10	4-chloro-*o*-toluidine (4-chloro-2-methylaniline)	202-441-6	95-69-2	Y
11	2-naphthylamine	202-080-4	91-59-8	N
12	aniline	200-539-3	62-53-3	N
13	benzidine	202-199-1	92-87-5	N
14	*p*-toluidine	203-403-1	106-49-0	N
15	2-methyl-*p*-phenylenediamine	202-442-1	95-70-5	N
16	biphenyl-4-ylamine	202-177-1	92-67-1	N
17	4-*o*-tolylazo-*o*-toluidine (*o*-aminoazotoluene)	202-591-2	97-56-3	Y
18	4-methoxy-*m*-phenylenediamine	210-406-1	615-05-4	N
19	4,4'-methylenedianiline (4,4′-diaminodiphenylmethane)	202-974-4	101-77-9	Y
20	4,4'-methylenedi-o-toluidine (4,4’-diamino-3,3'-dimethyldiphenylmethane)	212-658-8	838-88-0	Y
21	6-methoxy-*m*-toluidine (2-methoxy-5-methylaniline)	204-419-1	120-71-8	Y
22	4,4'-methylene-bis-(2-chloro aniline)	202-918-9	101-14-4	Y
23	4,4'-oxydianiline	202-977-0	101-80-4	Y
24	2,4,5-trimethylaniline	205-282-0	137-17-7	Y
25	4-Aminoazobenzene	200-453-6	60-09-03	Y
26	*p*-phenylenediamine	203-404-7	106-50-3	N
27	sulphanilic acid	204-482-5	121-57-3	N
28	4-amino-3-fluorophenol	402-230-0	399-95-1	Y
29	2,6-xylidine	201-758-7	87-62-7	Y
30	6-amino-2-ethoxynaphthaline (2-amino-6-ethoxynaphthalene)	-	293733-21-8	Y
31	2,4-xylidine	202-440-0	95-68-1	Y

Table S2. Recovery rates of three target aromatic amines (AAs) across different extraction solvent compositions, determined by GC–MS/SIM analysis of matrix-spiked 'Black1' samples.

Analytes	Recoveries according to extraction solvent composition (n = 3)		
	5:5 MeOH:DCM	3:7 MeOH:DCM	1:9 MeOH:DCM
o-Toluidine	69	75	92
2,4-Xylidine	79	85	97
2-Methoxy-5-methylaniline	77	88	105

Table S3. System suitability parameters for the 21 target AAs, including theoretical plate count (*N*), capacity factor (*k*), and injection repeatability (%RSD of peak area, $n = 3$). These parameters were calculated from the chromatograms of a mixed standard solution (0.1 mg/mL for each analyte) acquired using GC-MS in SIM mode.

ID No.	Analytes	CAS No.	*N*	*k*	Injection Repeatability (%RSD, $n = 3$)
1	*o*-Toluidine	95-53-4	4.06×10^5	5.79	1.6
2	2,4-Xylidine	95-68-1	6.28×10^5	6.71	1.6
3	2,6-Xylidine	87-62-7	6.25×10^5	6.77	1.0
4	*o*-Anisidine	90-04-0	6.29×10^5	6.98	1.2
5	4-Chloroaniline	106-47-8	5.78×10^5	7.35	2.2
6	4-Amino-3-fluorophenol	399-95-1	5.64×10^5	7.83	1.9
7	2-Methoxy-5-methylaniline	120-71-8	8.36×10^5	7.90	3.0
8	2,4,5-Trimethylaniline	137-17-7	9.00×10^5	7.96	2.1
9	4-Chloro-2-methylaniline	95-69-2	9.55×10^5	8.30	2.7
10	2-Methyl-5-nitroaniline	99-55-8	1.09×10^6	11.54	4.8
11	2-Amino-6-ethoxynaphthalene	293733-21-8	2.29×10^6	13.62	2.5
12	4-Aminoazobenzene	60-09-3	2.57×10^6	15.08	3.3
13	4,4′-Oxydianiline	101-80-4	2.07×10^6	15.54	2.4
14	4,4′-Diaminodiphenylmethane	101-77-9	2.18×10^6	15.61	3.1
15	*o*-Aminoazotoluene	97-56-3	2.06×10^6	16.29	2.6
16	4,4’-Diamino-3,3'-dimethyldiphenylmethane	838-88-0	1.82×10^6	16.94	2.5
17	*o*-Tolidine	119-93-7	1.53×10^6	17.27	2.4
18	4,4′-Thiodianiline	139-65-1	1.00×10^6	18.53	3.6
19	4,4’-Methylenebis(2-chloroaniline)	101-14-4	1.19×10^6	19.05	2.1
20	3,3’-Dichlorobenzidine	91-94-1	1.09×10^6	19.09	2.9
21	*o*-Dianisidine	119-90-4	8.57×10^5	19.17	3.7

Table S4. Evaluation of matrix effects (ME) for AAs in the 'Black1' tattoo ink sample. ME was determined using the formula: ME = (m_2/m_1) × 100 (%) where m_1 and m_2 represent the slopes of calibration curves obtained from solvent standards and matrix-matched standards (Black1 ink extract), respectively.

ID No.	Analytes	ME = (m_2/m_1) × 100 (%)
1	*o*-Toluidine	100
2	2,4-Xylidine	93
3	2,6-Xylidine	120
4	*o*-Anisidine	119
5	4-Chloroaniline	106
6	4-Amino-3-fluorophenol	131
7	2-Methoxy-5-methylaniline	108
8	2,4,5-Trimethylaniline	111
9	4-Chloro-2-methylaniline	106
10	2-Methyl-5-nitroaniline	115
11	2-Amino-6-ethoxynaphthalene	113
12	4-Aminoazobenzene	104
13	4,4′-Oxydianiline	92
14	4,4′-Diaminodiphenylmethane	90
15	*o*-Aminoazotoluene	106
16	4,4'-Diamino-3,3'-dimethyldiphenylmethane	91
17	*o*-Tolidine	92
18	4,4′-Thiodianiline	99
19	4,4'-Methylenebis(2-chloroaniline)	103
20	3,3'-Dichlorobenzidine	102
21	*o*-Dianisidine	105

Table S5. Evaluation of cross-matrix applicability: Recoveries (%) of AAs in various colored tattoo inks. Note: Recovery values (±%RSD) were quantified against the 'Black1' matrix-matched calibration curve. Data represent technical replicates (*n* = 3 injections of a single fortified extract per color).

ID No.	Analytes	Red	Blue	Green	Yellow
1	*o*-Toluidine	104±0.4	109±0.4	101±0.7	104±1.0
2	2,4-Xylidine	107±0.4	100±0.2	103±0.5	106±0.7
3	2,6-Xylidine	114±0.3	105±0.2	110±0.3	111±0.7
4	*o*-Anisidine	108±0.5	102±0.2	106±0.4	108±0.7
5	4-Chloroaniline	74±1.4	101±0.5	104±0.6	107±1.3
6	4-Amino-3-fluorophenol	84±0.8	99±0.6	98±2.0	99±1.4
7	2-Methoxy-5-methylaniline	103±0.4	103±0.4	107±0.6	109±0.9
8	2,4,5-Trimethylaniline	95±0.8	102±1.0	93±0.6	94±0.7
9	4-Chloro-2-methylaniline	103±0.6	100±0.6	103±0.5	106±1.4
10	2-Methyl-5-nitroaniline	93±0.6	94±1.4	94±3.0	99±1.7
11	2-Amino-6-ethoxynaphthalene	89±1.2	94±0.9	86±2.0	84±1.6
12	4-Aminoazobenzene	100±0.5	104±0.1	89±4.3	90±1.1
13	4,4′-Oxydianiline	78±4.5	85±3.5	83±4.9	86±3.9
14	4,4′-Diaminodiphenylmethane	92±2.3	90±1.0	97±2.4	91±0.5
15	*o*-Aminoazotoluene	101±0.4	106±0.2	109±0.4	111±0.8
16	4,4'-Diamino-3,3'-dimethyldiphenylmethane	86±2.0	98±0.5	102±0.4	103±0.4
17	*o*-Tolidine	91±0.5	102±1.1	106±0.4	107±0.3
18	4,4′-Thiodianiline	82±1.1	93±1.2	100±0.6	96±0.8
19	4,4'-Methylenebis(2-chloroaniline)	103±0.1	109±0.8	112±0.2	92±0.7
20	3,3'-Dichlorobenzidine	101±0.4	109±0.9	112±0.9	88±0.4
21	*o*-Dianisidine	90±1.0	100±0.6	104±1.5	101±1.8

Table S6. Comparative evaluation of relative matrix effects (ME) for AAs in various colored tattoo inks relative to the 'Black1' matrix. Note: ME was determined using the formula: ME = (m_2/m_1) × 100 (%), where m_1 and m_2 represent the slopes of the calibration curves obtained from the 'Black1'-matrix matched standards and the respective colored (Red, Blue, Green, or Yellow) matrix-matched standards, respectively.

		ME = (m_2/m_1) × 100 (%)			
ID No.	Analytes	Red	Blue	Green	Yellow
2	2,4-Xylidine	94	112	118	118
5	4-Chloroaniline	107	92	98	97
7	2-Methoxy-5-methylaniline	100	95	101	100
9	4-Chloro-2-methylaniline	102	98	105	104
10	2-Methyl-5-nitroaniline	102	95	99	103
12	4-Aminoazobenzene	99	89	109	113
15	*o*-Aminoazotoluene	100	92	96	97
17	*o*-Tolidine	97	102	107	103
19	4,4'-Methylenebis(2-chloroaniline)	100	89	93	112
20	3,3'-Dichlorobenzidine	102	86	89	110

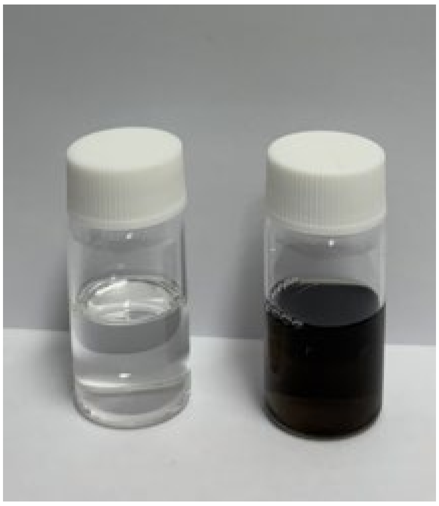

Figure S1. Visual comparison of filtrates from ‘Black1’ tattoo ink (50-fold diluted with 1:9 MeOH:DCM) after filtration through a hydrophilic PTFE filter (left) and a hydrophobic PTFE filter (right).

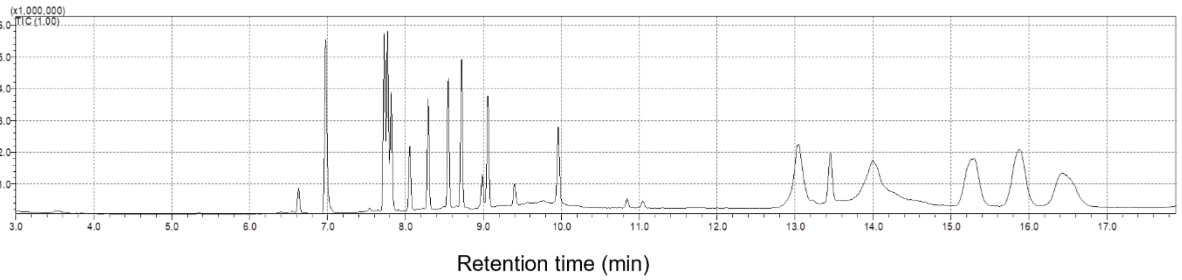

Figure S2. GC-MS chromatogram of target ions of 21 AAs with a DB-624 column acquired in SIM mode.

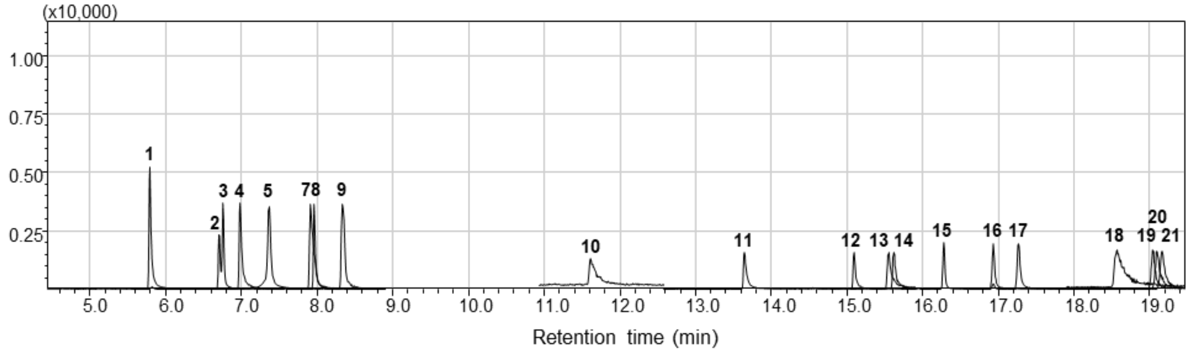

Figure S3. MRM chromatograms for AAs at a concentration of 5 mg/kg in the 'Black1' matrix. Indicated numbers on peaks correspond to ID numbers of AAs listed in Table 3.

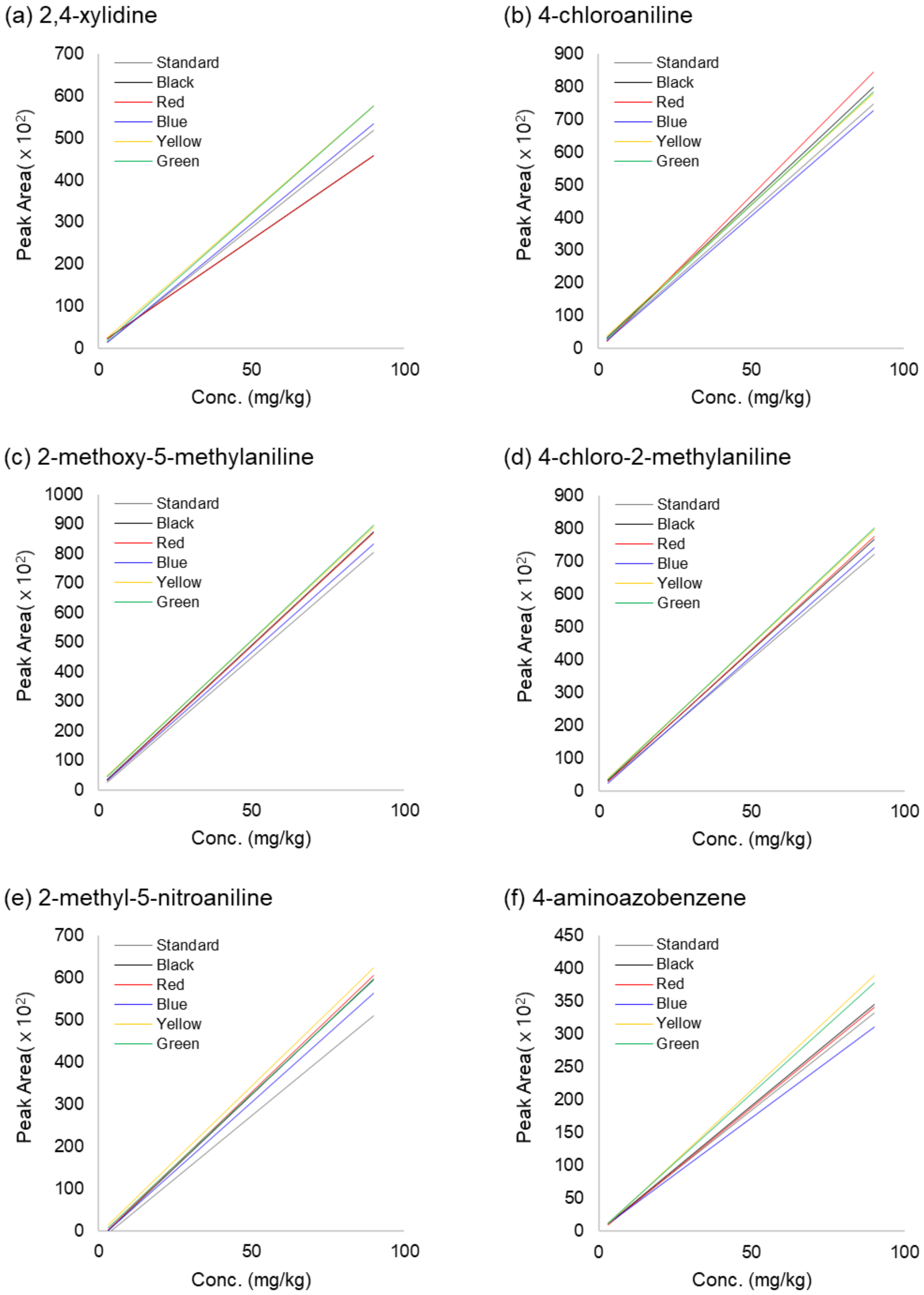

Figure S4. Standard and matrix-matched calibration curves of ten representative AAs across different colored tattoo ink matrices (Black1, Red, Blue, Yellow, and Green) and solvent standard: (a) 2,4-xylidine, (b) 4-chloroaniline, (c) 2-methoxy-5-methylaniline, (d) 4-chloro-2-methylaniline, (e) 2-methyl-5-nitroaniline, (f) 4-aminoazobenzene, (g) *o*-aminoazotoluene, (h) *o*-tolidine, (i) 4,4'-methylenebis(2-chloroaniline), and (j) 3,3'-dichlorobenzidine.

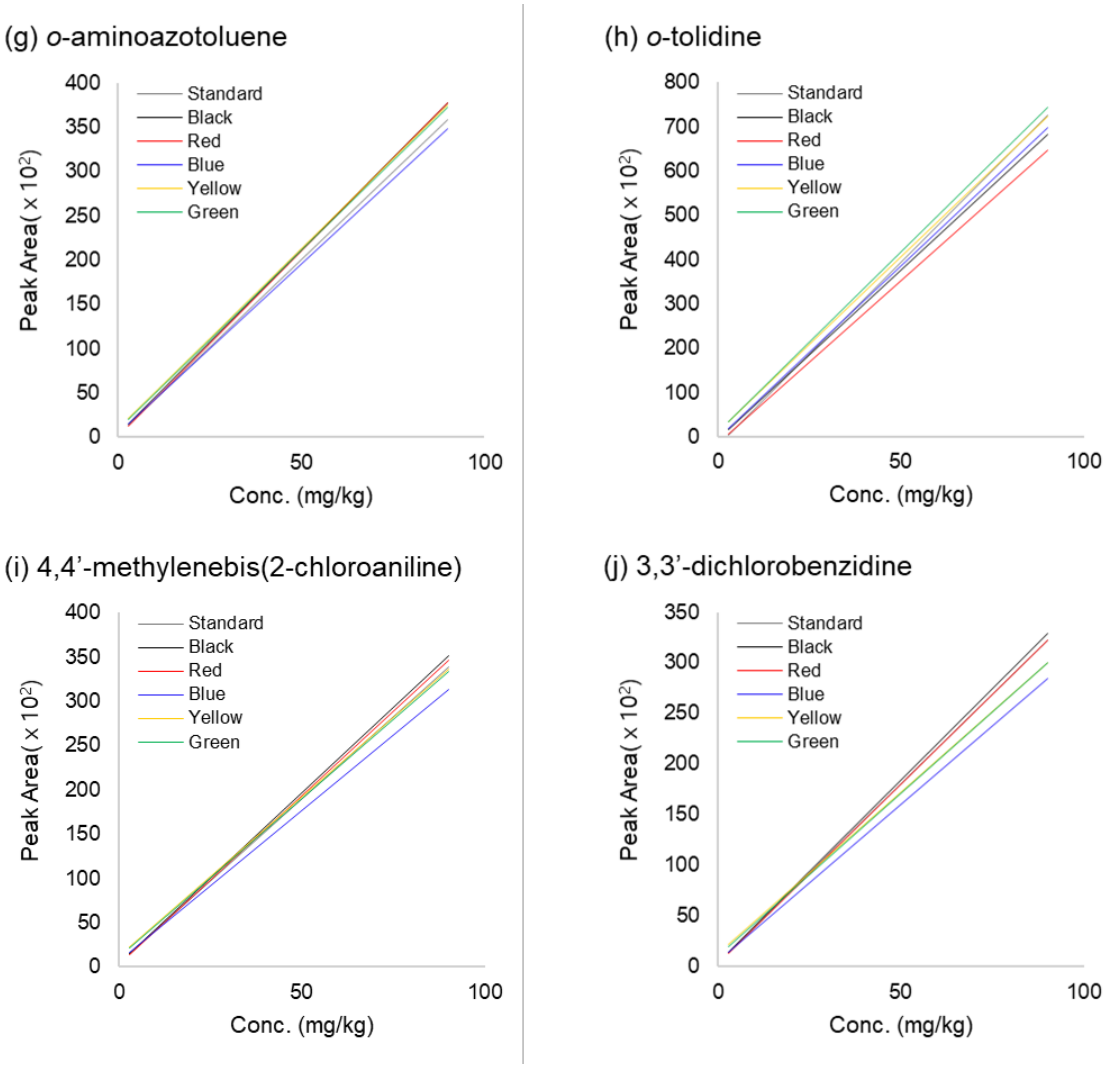

Figure S4 (*Continued*).

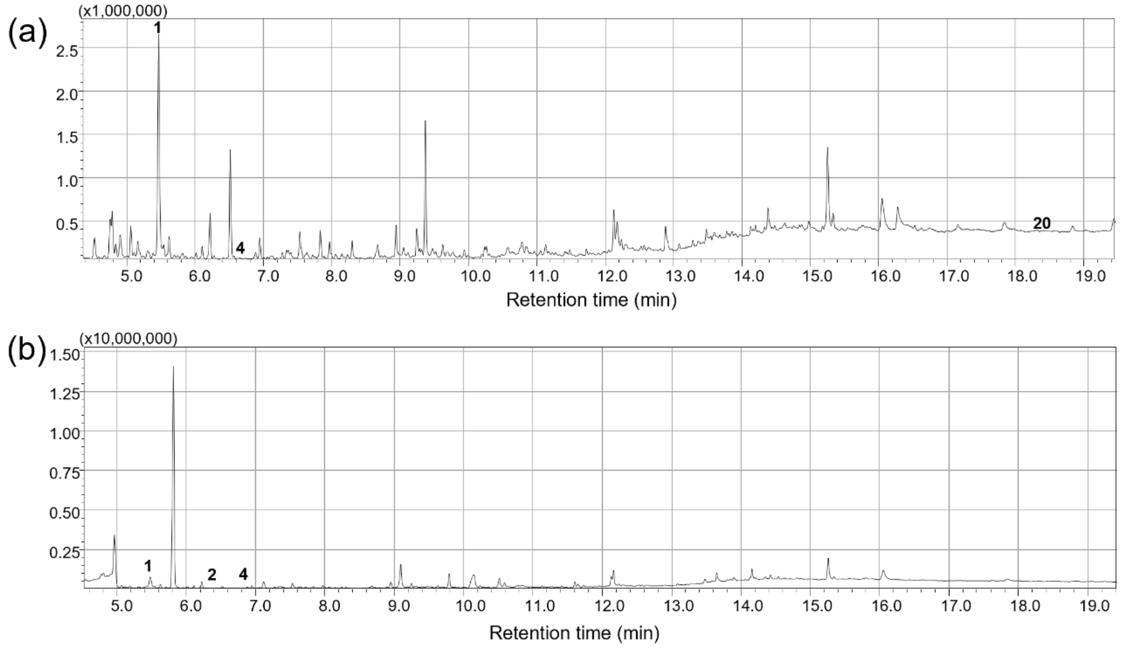

Figure S5. GC-MS chromatograms of (a) 'Green' and (b) Yellow' tattoo inks acquired in full scan mode. Indicated numbers on peaks correspond to ID numbers of AAs listed in Table 6.

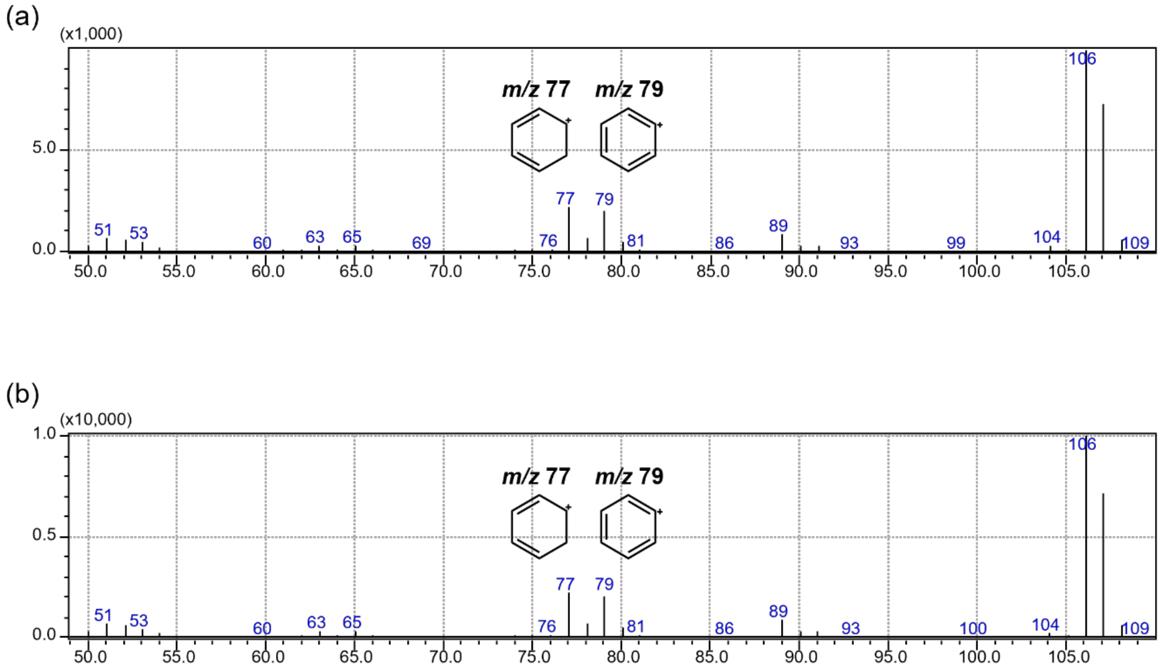

Figure S6. (a) EI mass spectrum of the o-toluidine standard solution and (b) EI mass spectrum extracted from the GC-MS analysis of the 'Green' tattoo ink sample.

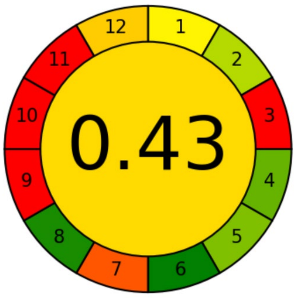

Figure S7. AGREE-based greenness assessment of the proposed method.

References for AGREE-based greenness assessment:

Anal. Chem. 2020, 92, 14, 10076–10082, https://doi.org/10.1021/acs.analchem.0c01887
